# Axillary lymphadenectomy for breast cancer in elderly patients and fibrin glue

**DOI:** 10.1186/1471-2482-13-S2-S8

**Published:** 2013-10-08

**Authors:** Giovanni Docimo, Paolo Limongelli, Giovanni Conzo, Simona Gili, Alfonso Bosco, Antonia Rizzuto, Vincenzo Amoroso, Salvatore Marsico, Nicola Leone, Antonio Esposito, Chiara Vitiello, Landino Fei, Domenico Parmeggiani, Ludovico Docimo

**Affiliations:** 1 Division of General Surgery, Department of Surgery, Second University of Naples, Via S. Pansini 5, 80131, Naples, Italy; 2 Department of Medical and Surgical Science, Magna Grecia University, Catanzaro, Italy

## Abstract

**Background:**

Axillary lymphadenectomy or sentinel biopsy is integral part of breast cancer treatment, yet seroma formation occurs in 15-85% of cases. Among methods employed to reduce seroma magnitude and duration, fibrin glue has been proposed in numerous studies with controversial results.

**Methods:**

Thirty patients over 60 years underwent quadrantectomy or mastectomy with level I/II axillary lymphadenectomy; a suction drain was fitted in all patients. Fibrin glue spray were applied to the axillary fossa in 15 patients; the other 15 patients were treated with harmonic scalpel.

**Results:**

Suction drainage was removed between post-operative Days 3 and 4. Seroma magnitude and duration were not significant in patients receiving fibrin glue compared with the harmonic scalpel group.

**Conclusions:**

Use of fibrin glue does not always prevent seroma formation, but can reduce seroma magnitude, duration and necessary evacuative punctures.

## Background

Axillary dissection represents an integral part of the treatment for breast cancer for prognostic and curative purposes. There is still a significant incidence (15-81%) of complications associated with axillary lymphadenectomy, including lymphorrhea, lymphoceles, and in rare cases lymphedema (swelling of the arm) [1-5]. It is possible to avoid axillary dissection in selected patients (T1 N0) using the sentinel lymph node technique. However, in all cases where T > 3 cm, and in patients with T1 N1 or with a metastatic sentinel lymph node, conventional axillary lymphadenectomy remains the primary surgical protocol for quadrantectomy and mastectomy. Many different methodologies have been used in an effort to reduce seroma formation following axillary lymphadenectomy for breast cancer. These include suction drainage [6,7] topical application of tetracycline [8], closing and stitching the axillary fossa [9,10], axillary dissection with axilloscopy [11], external compression [12,13], use of harmonic scalpel and application of fibrin glue [14,15]. To date, no practical guidelines exist on how to conduct suction drainage, and the views of surgeons are varied. Some surgeons believe that it is best to remove the drain on the first day of the post-operative period, some think the drain should be left in place until the drained volume falls below 50 ml/day [16], while others feel that drainage could in fact prolong and intensify the inflammatory stage of the wound-healing process, with a subsequent increase in seroma formation [17]. Regardless of contradicting opinions, the possibility of reducing the time for which drainage is present during the recovery period, therefore eliminating the need to discharge patients with drainage *in situ*, may represent a valid solution for reducing seroma formation. This prospective study evaluates the effectiveness of using fibrin glue to reduce seroma formation following axillary lymphadenectomy for breast cancer.

## Methods

Between January 2010 and December 2012, 30 patients over 60 years were enrolled in the study. All patents had N+ breast adenocarcinoma and required either a quadrantectomy or a modified radical mastectomy with level I or II axillary lymphadenectomy, extending from the lower border of the axillary vein superiorly, from the medial border of the smaller pectoral muscle medially, as far as the fourth intercostobrachial nerve inferiorly and to the edge of the latissimus dorsi laterally. The axillary lymphadenectomy was performed in 15 patients with ligations, scissors and electrocautery and in 15 patients with ultrasound scalpel [18]. To be enrolled in the study, patients were required to have no alterations in their blood clotting or immune systems, or at least not be receiving anticoagulant treatment; have no history of sensitisation to bovine aprotinin; no psychological changes; no uncompensated diabetes or advanced liver disease; not be severely obese, and not have had previous surgery on the axillary lymphatic system or any immediate reconstructive surgery. Short-term antibiotic prophylaxis was applied. After surgery, all patients were fitted with a suction drain at the axillary fossa, which was activated 10 minutes after stitching the skin. The harmonic scalpel group received the suction drain without additional treatment, while the other group received fibrin glue (Tisseal^®^/Tissucol^®^, Baxter Healthcare Corporation, Deerfield, IL, USA). For patients in the fibrin glue treatment group, following fitting of the drain, fibrin glue prepared with 500 IU/ml of thrombin was applied as a spray (2 ml, from a distance of 10 cm with 2 bars of pressure) at the site of the quadrantectomy or mastectomy. Fibrin glue prepared with 5 IU/ml of thrombin was also applied as a spray (2 ml) to the axillary fossa with approximately three minutes' manual compression. A compression dressing was applied over the treatment site and kept in place for at least 24-72 hours. Fibrin glue for the axillary fossa was prepared by diluting it to 5 IU/ml with calcium chloride solution (20 mM), a concentration capable of maintaining activation of the wound-healing process through a thick mesh of fibrin fibrils. Patients were discharged when the volume drained fell below 100 ml/day and suction drainage could be removed. One week later, patients underwent clinical and instrumental (ultrasound) assessment for the presence of seroma, and any residue was suctioned out using an echo-guided evacuative puncture, as necessary. The primary outcome measures of the study were a) the incidence of seroma, including total volume of serum drained, and b) number of evacuative punctures required. A secondary outcome measure of the study was the duration of the treatment up to the time of healing. Complications associated with the axillary lymphadenectomy procedure were also recorded.

Statistical analysis was performed on the patients who completed the postoperative evaluation using SPSS for Windows (version 17.0; SPSS, Inc., Chicago, IL). Results were expressed as mean ± SD unless otherwise indicated. Continuous variables were compared by Student *t *test and dichotomic variables were compared by the chi-squared test and the McNemar test. P value < 0.05 was considered statistically significant.

## Results

Factors as age, size of tumors and its ratio to the residual breast volume following surgery, the number of lymph node removed and the number of metastatic lymph nodes, also did not represent significant variables in terms of seroma magnitude. All patients were discharged 3-4 days following surgery. Compared with patients in the control group, patients in the fibrin glue group had a lower drainage volume 150 [60-350] *versus *180 [110-400] ml; *p *= NS), fewer evacuative punctures (2 [0-3] *versus *3 [1-3]), and a not significantly reduced mean seroma duration i.e. healing time 12 [4-21]*versus *14 (4-25) days; *p *= NS). The incidence of complications was 20% (3 with cellulitis) and 20% (2 with cellulitis and 1 with wound diastasis) in patients who underwent surgery with and without fibrin glue, respectively. No significant relationship was observed in either patient group, between the magnitude of seroma formation and the type of surgical procedure (quadrantectomy or mastectomy) conducted.

## Discussion

Breast cancer surgery, and in particular axillary lymphadenectomy, has changed in the last few years with the advent of the sentinel lymph node technique [11]. However, even today there are a number of situations where a conventional axillary lymphadenectomy is indicated, including patients with tumours greater than 3 cm in diameter, with positive or suspect axillary lymph nodes based on an objective examination and an instrumental diagnosis, or with a positive sentinel lymph node. In this group of patients, the axillary lymphadenectomy still has complications, in particular seroma formation (15-81%)[1-5], which can delay the patient's discharge, healing and supplementary radiotherapy and chemotherapy treatments. The formation of seroma can result from a lesion of the axillary lymphatic vessels [2] or from an inflammatory reaction [17], which may also be prolonged and intensified by the continuance of suction drainage. The removal of drainage for some surgeons is indicated when the volume per day is less than 50 ml^2^, but we prefer to remove the drainage when the volume per day is less than 100 ml, since in our experience drains do not prevent seroma formation, and they also dictate the date of discharge, resulting in a longer stay [3]. A number of surgeons believe that it is possible to discharge patients with the drainage *in situ *[3] despite the associated discomfort and increase in percentage infection rate. This issue of drainage management in the home arises in cases of procedures carried out in day surgery. Many published articles on the usefulness of drainage following axillary lymphadenectomy contradict each another with regard to seroma control, magnitude and duration. Porter^17^reported a non-significant difference in the incidence and degree of seroma between patients with suction drainage (73%) *versus *patients without suction drainage (89%). Divino [4] reported a 6% incidence of seroma for patients with drainage, compared with 40% for patients without. Burak [19] noted a relationship between seroma magnitude and patient age. Jeffrey [20] reported a 92% seroma incidence in patients without drainage, and sharing the view of others that repeated suctions may be the cause of infections of the axillary fossa [21-25], applied evacuative puncture only in symptomatic cases (42%). In terms of the methods of reducing seroma magnitude, there have been numerous reports of the benefits of using an external compression dressing [26], immobilization of the arm [27], use of sutures to close the axillary fossa [28], excessive use of the electric scalpel compared to ligature of the lymphatic branches [29,30], benefits of multiple drains[21], and the type of suction (high- or low-pressure) applied [31]. The use of fibrin glue has also produced contrasting results: reduction of seroma according to Moore and Gilly [11,26] no difference in seroma formation compared with patients treated without fibrin glue, according to Burak [19], Langer [27] and Dinsmore [28]. The latter Authors attributed the lack of benefit to the presence of drainage possibly interfering with the stabilization of a fibrin clot, and with closure of the lymphatic capillaries. Fibrin glue interacts with the tissues damaged during the surgical procedure, favoring the growth of fibroblasts and wound healing. It favors hemostasis by preventing hematomas, which delay the surgical healing processes; makes the lymphatic branches impermeable, reducing seroma formation; and makes it possible to close the dead spaces through tissue adhesion. A number of papers have presented comparative studies of patients with and without fibrin glue in the axillary fossa. In a study of 24 patients who underwent quadrantectomy or mastectomy with axillary lymphadenectomy, In a group of 20 patients who underwent quadrantectomy with axillary lymphadenectomy, Jain [29] reported a seroma incidence of around 40%, with a reduction in seroma magnitude in mastectomy patients upon whom fibrin glue was used. Although a significant difference was not observed between quadrantectomy patients treated with or without fibrin glue. The study demonstrated that suction drainage did not limit the incidence or magnitude of seromas, and that it was associated with extended time spent in hospital and post-operative pain. Soon [30] showed that, among patients who underwent quadrantectomy or mastectomy with axillary lymphadenectomy, there was no difference in terms of the incidence of seromas with or without the use of suction drainage, and that, for the group of patients without drainage, the seromas formed in greater magnitude and for a longer duration, but with a lower percentage of complications. Kuroi [31] in a Medline search about one meta-analysis, 51 randomized controlled trials, 7 prospective studies and 7 retrospective studies showed that there was moderate evidence to support a risk for seroma formation in individuals with heavier body weight, extended radical mastectomy and greater drainage volume in the initial 3 days; with regard to the use of adhesive glue many retrospective studies failed to show any significant effect on seroma formation. Based on our experience and reviewing data from the literature [3,10,21,31-37], it seems that the magnitude and duration of the seromas are limited, but they are present in over 80% of patients, without significant differences between mastectomy and quadrantectomy. Use of harmonic scalpel can reduce the magnitude of seromas and acute blood loss after axillary dissection [38-48]. The use of fibrin glue may therefore be useful, in our opinion, in traditional breast cancer surgery for reducing seroma magnitude and duration, and shortening the stay in hospital which, in this pathology too, is increasingly conducted in day surgery.

## Competing interests

The authors declare that they have no competing interests.

## Authors' contributions

GD, PL, DP have made substantial contributions to conception and design; SG, AB have made substantial contributions to acquisition of data; AR, VA, have made substantial contributions to analysis and interpretation of data; NL, AE, have been involved in drafting the manuscript; PL, DP, LD have been involved in revising it critically for important intellectual content; GD, PL, DP and LD have given final approval of the version to be published.

## Authors' information

GD Associate Professor of Surgery at Second University of Naples; PL Assistant Professor of Surgery at Second University of Naples; GC Assistant Professor of Surgery at Second University of Naples; SG Research Fellow at Second University of Naples; AB Surgical Fellow at Second University of Naples; AR Research Fellow at Magna Grecia University; VA Surgical Fellow at Second University of Naples; SM Medical Student at Second University of Naples; NL Medical Student at Second University of Naples; AE, Medical Student at Second University of Naples; CV Medical Student at Second University of Naples; LF Full Professor of Surgery at Second University of Naples; DP Assistant Professor of Surgery at Second University of Naples; LD Full Professor of Surgery at Second University of Naples.

## Declarations

This article has been funded by personal funds.

This article has been published as part of *BMC Surgery *Volume 13 Supplement 2, 2013: Proceedings from the 26th National Congress of the Italian Society of Geriatric Surgery. The full contents of the supplement are available online at http://www.biomedcentral.com/bmcsurg/supplements/13/S2
